# A Novel Mutation in the *EIF2B4* Gene Associated with Leukoencephalopathy with Vanishing White Matter

**DOI:** 10.1155/2018/2731039

**Published:** 2018-07-05

**Authors:** D. Hettiaracchchi, N. Neththikumara, B. A. P. S. Pathirana, A. Padeniya, V. H. W. Dissanayake

**Affiliations:** ^1^Human Genetics Unit, Faculty of Medicine, University of Colombo, Colombo, Sri Lanka; ^2^National Hospital of Sri Lanka, Colombo, Sri Lanka

## 1. Introduction

Leukoencephalopathy with vanishing white matter (VWM; MIM #603896) is an autosomal recessive disorder, characterized by childhood ataxia, spasticity, and variable optic atrophy. The course is chronic progressive with episodes of rapid deterioration, provoked by febrile illnesses, minor head trauma, or acute fright, with most patients succumbing to illness within few years of onset. Three forms of VWM has been described based on disease onset, which ranges from a subacute infantile form (onset age <1 year), an early childhood form (onset age 1–5 years), and a late-childhood/juvenile form (onset age 5–15 years) [1–3]. The diagnosis is based on clinical findings, characteristic MRI features indicative of vanishing of the cerebral white matter, and an identifiable pathogenic variant in one of the genes (*EIF2B1*, *EIF2B2*, *EIF2B3*, *EIF2B4*, and *EIF2B5*) encoding for the 5 subunits of eukaryotic translation initiation factor 2B (eIF2B), which is essential in all cells of the body for the initiation of translation of RNA into protein during protein synthesis and its regulation under different stress conditions such as fever [4, 5]. Its effect is predominantly seen on oligodendrocytes and astrocytes while there is sparing of other cell types [2].

## 2. Case Report

**Figure 1:**
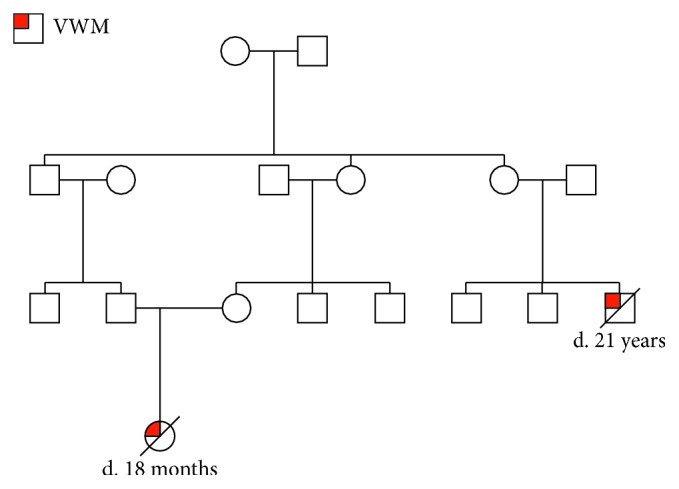
Pedigree chart of the proband with affected family members.

The proband is the only child of a 2nd degree consanguineous marriage of Sri Lankan origin. She was well up to 8 months when she developed a fever for one day, following which the child had acute developmental regression that lasted for 3 months accompanied with bilateral lower limb weakness and speech regression. The child then developed an upper respiratory tract infection, following which she was unresponsive for 30 minutes. The MRI scan showed marked hyperintense and subcortical white matter in T2 WI bilaterally with involvement of dentate nuclei and white matter tracts. Myelination was around 3 months, which corresponded to the current developmental age of the child. All biochemical parameters were within normal limits. The proband succumbed to illness at 18 months. She also had a first cousin with similar features who died at the age of 21 years (Figure 1).

## 3. Methods

### 3.1. Whole Exome Sequencing

DNA was extracted from the proband's whole blood leucocytes using Qiagen DNA extraction Mini KIT according to the manufacturer's protocol. Whole exome sequencing (WES) of the extracted DNA was performed on an Illumina® HiSeq 4000 Next Generation Sequencer using the SureSelect® Human All Exon V6 kit.

### 3.2. Bioinformatics Analysis

Data analysis was performed using an in-house-developed variant calling annotation pipeline. Paired-end Fastq files were aligned to the GrCh37 human reference sequence using BWA-MEM algorithm to produce SAM file. The SAM to BAM conversion, sorting, and indexing were performed using SAM tools, and deduping of reads were performed using Picard tools. Variant discovery was performed using the Genome Analysis Tool Kit (GATK) Haplotype caller, followed by realigning of the deduped BAM around indels. Annotation of the resulted VCF file was performed using SNP-eff with Refseq, dbSNP, 1000 Genomes, Exome Variant Server, Exome Aggregation Consortium, phastCons100way, ClinVar, and locus-specific databases. MutationTaster, SIFT, PolyPhen2, and Provean were used to in silico functional prediction. Reported benign variants resided in the genes causing VWM were filtered out using virtual gene panel. Remaining variants were further scrutinized considering their availability in public databases, their conservation, and their functional impact on the protein.

## 4. Results

**Figure 2:**
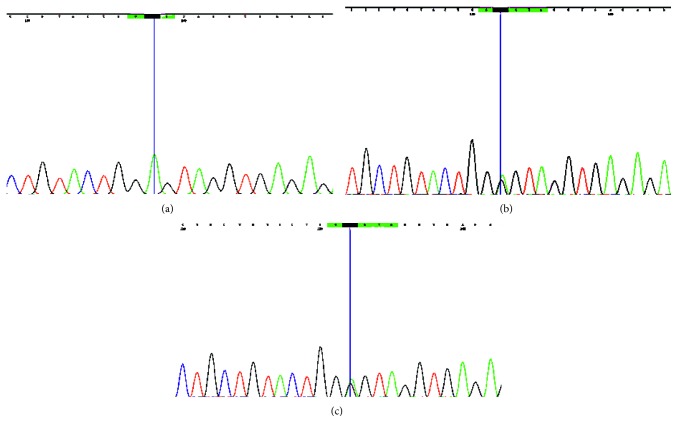
(a) Sanger sequence chromatogram of the proband showing a novel homozygous missense mutation in exon 5 of the eukaryotic translation initiation factor 2B subunit delta (*EIF2B4*) gene, ENST00000493344: c.614C>T [p.Pro205Leu] causing leukoencephalopathy with vanishing white matter (VWM). (b) Mother of the proband with a heterozygous mutation. (c) Father of the proband with a heterozygous mutation.

A novel missense mutation in exon 5 of the eukaryotic translation initiation factor 2B subunit delta gene (*EIF2B4*), ENST00000493344: c.614C>T| p.Pro205Leu was detected (read depth: 60x). The patient was a homozygote for the mutation. Mutation was also seen in the Sanger sequence chromatogram. (Figure 2) p.Pro205Leu was a novel mutation and therefore is absent in population genetic databases and clinical databases. It results in a nonconservative amino acid substitution, which impacts the physiochemical nature of the protein. This mutation resides in a highly conserved region among different species through out the evolution (phastCons100way_vertebrate score = 1). In silico prediction tools universally concluded that p.Pro205Leu has a deleterious effect (Table 1) on protein structure and function.

## 5. Discussion

Vanishing white matter disease (VWM) is autosomal recessive leukodystrophy linked to mutations in translation initiation factor 2B (eIF2B), and it is the only brain disease recognized to date, which involves this initiation factor. Reduction in eIF2B activity leads to a cascade of cellular events resulting in a sustained improper activation of the unfolded protein response and concomitant expression of proliferation, prosurvival, and proapoptotic downstream effectors. These events predispose VWM cells to stress-induced hyperreactive damage [6–8]. Even though the cellular consequences of *EIF2B* mutations on neural cells are unknown, cell cultures from the brain of an individual with VWM carrying mutations in subunit 5 of eIF2B (encoded by *EIF2B5*) have generated morphologically normal oligodendrocytes and abnormal astrocytes *in vitro*, suggesting that a deficiency in astrocyte function may contribute to the loss of white matter in VWM leukodystrophy [6].

The resulting pathological manifestations include increasing white matter rarefaction and cystic degeneration, oligodendrocytosis with highly characteristic foamy oligodendrocytes, meager astrogliosis with dysmorphic astrocytes, and loss of oligodendrocytes by apoptosis, but their exact pathophysiology is poorly understood [7, 9, 10]. Currently, treatment strategies involving neural stem cell transplantation are being explored, but their robust proliferative capacity coupled with difficulty to control their differentiation potential remains the main challenge for cell-based therapy, as these transplanted cells can potentially generate undesired neural cell types [11, 12]. Due to astrocytes being the predominantly affected cell type, transplantation of immature astrocytes might have a theoretical benefit.

The high incidence of low-frequency mutations limits rapid genetic confirmation of the disease, especially in cases of undiagnosed leukodystrophy [13, 14]. In our patient, the P205L was a novel mutation and therefore is absent in population genetic databases and clinical databases. It results in a nonconservative amino acid substitution, which impacts the physiochemical nature of the protein. We can conclude that all in silico tools predict a deleterious impact on protein function, hence giving rise to the phenotypic features of VWM leukodystrophy. By reporting this novel mutation, we wish to facilitate its early detection.

## Figures and Tables

**Table 1:** Results of in silico mutation prediction analysis.

Algorithm	Prediction	Score
PolyPhen2^a^	Probably damaging (HumDiv- model)	0.998
PolyPhen2^a^	Probably damaging (HumVar- model)	0.980
Provean^b^	Deleterious	−4.92
SIFT^c^	Damaging	0.032
MutationTaster^d^	Disease causing	0.999

^a^ http://genetics.bwh.harvard.edu/pph2; deleterious threshold >0.5. ^b^http://provean.jcvi.org/index.php; score threshold is −2.5 for binary classification. ^c^http://sift.jcvi.org/www/SIFT_chr_coords_submit.html threshold <0.05. ^d^http://www.mutationtaster.org; scores range from 0.0 to 1.0.

## Additional Points

*Key Clinical Message*: Leukoencephalopathy with vanishing white matter is an autosomal recessive disorder characterized by childhood ataxia, spasticity, and variable optic atrophy. Pathological variants in one of the five genes encoding the five subunits of eukaryotic translation initiation factor eIF2B has been implicated. The authors report a novel homozygous variant in *EIF2B4* gene.
